# The role of codon selection in regulation of translation efficiency deduced from synthetic libraries

**DOI:** 10.1186/gb-2011-12-2-r12

**Published:** 2011-02-01

**Authors:** Sivan Navon, Yitzhak Pilpel

**Affiliations:** 1 Department of Molecular Genetics, Weizmann Institute of Science, PO Box 26, Rehovot, 76100, Israel

## Abstract

**Background:**

Translation efficiency is affected by a diversity of parameters, including secondary structure of the transcript and its codon usage. Here we examine the effects of codon usage on translation efficiency by re-analysis of previously constructed synthetic expression libraries in *Escherichia coli*.

**Results:**

We define the region in a gene that takes the longest time to translate as the bottleneck. We found that localization of the bottleneck at the beginning of a transcript promoted a high level of expression, especially if the computed dwell time of the ribosome within this region was sufficiently long. The location and translation time of the bottleneck were not correlated with the cost of expression, approximated by the fitness of the host cell, yet utilization of specific codons was. Particularly, enhanced usage of the codons UCA and CAU was correlated with increased cost of production, potentially due to sequestration of their corresponding rare tRNAs.

**Conclusions:**

The distribution of codons along the genes appears to affect translation efficiency, consistent with analysis of natural genes. This study demonstrates how synthetic biology complements bioinformatics by providing a set-up for well controlled experiments in biology.

## Background

Understanding the mechanisms that control the efficiency of protein translation is a major challenge for proteomics, computational biology and biotechnology. Efficient translation of proteins, either in their natural biological context or in heterologous expression systems, amounts to maximizing production, while minimizing the costs of the process. Abundant genome sequence data now make it possible to decipher sequence design elements that govern the efficiency of translation. The codon adaptation index (CAI) [1] was the first measure to be introduced for gauging translation efficiency directly from nucleotide sequences of genes. This measure quantifies the extent to which the codon bias of a gene resembles that of highly expressed genes. The tRNA adaptation index (tAI) assesses the extent to which the codons of a gene are biased towards the more abundant tRNAs in the organism [2]. Despite several simplifying assumptions, both tAI and CAI are good measurements for predicting protein abundance from sequence [3,4]. Perhaps the most critical simplification of the two models is that they represent the translation efficiency of an entire gene by a single number - the average translation efficiency value over all its codons. As such, both CAI and tAI ignore the order in which codons of high and low translation efficiency appear in the sequence. Thus, two genes may share the same value of CAI or tAI and yet the order of high and low efficiency codons differs between them.

By analyzing dozens of genomes, we have recently shown that the order of high and low efficiency codons in biological sequences is under selection [5,6]. Specifically, examining such genomes revealed a clustering of low efficiency codons at the beginning of ORFs, mainly in the first approximately 50 codons. We termed this design the 'translation ramp', or 'ramp' for short, which might constitute a strategic early bottleneck in the flow of the ribosomes. Our model suggests that such ramps attenuate the ribosomes at the beginning of genes, thus allowing a jam-free flow of ribosomes beyond the ramp. We have shown that this design is predominantly obeyed by highly expressed genes [5,7], suggesting that it might support efficient production. Investigating natural genes has two obvious advantages: their availability in very high numbers, and the fact that they have been subject to selection and optimization by evolution. Similarly, using the totally asymmetrical simple exclusion process (TASEP), it was theoretically shown that slow codons can affect ribosome density and production rates depending on initiation rate, termination rate, and the rate of the slow codons and their distribution [8-12].

Yet, analysis of natural sequences also poses limitations. Natural genes represent a wide variety but their variability is uncontrolled and is influenced by confounding factors at many levels. For instance, even if two genes share the same translation efficiency profile, they may differ with respect to the strength of their promoter, the un-translated regions, the secondary structure and the amino acid sequence, all factors that may affect protein levels. Synthetic biology, which now offers the ability to synthesize and express designed genes, may complement the picture obtained from bioinformatics analysis of natural genes. Although the number of genes that can be synthesized is by orders of magnitude lower than the number of natural sequences, synthetic genes enable us to modify one variable at a time while keeping others constant. In several pioneering studies of this type, the nucleotide sequence of a single gene was randomized while amino acid sequence was kept constant. In particular, these studies generated libraries of artificial variants of genes' nucleotide coding sequences, while fixing other features, such as the un-translation regions and promoters. Analysis of one such library led to an important finding - that the stability of the mRNA, especially in the 5' region, is a main determinant of protein abundance [13]. Those authors further found that the CAI of a gene had no effect on protein expression levels but that it was rather correlated with, and perhaps affected, the fitness of the host cell.

Here we set to re-analyze the data from these libraries [13,14]. We were motivated by the realization that, due to their simplifying assumptions, the CAI and tAI do not capture the full capacity of codon selection to affect translation efficiency, particularly since these models ignore codon order that is under tight selection [5,6]. We show that obeying the design we observed in nature, namely localization of the bottleneck at the beginning of the ORF sequence, indeed promotes higher levels of expression. This was especially true if the predicted dwell time of the ribosome at these bottleneck regions was sufficiently long. On the other hand, the bottleneck characteristics did not affect the fitness of the host cell. We did find, however, that the extent of utilization of two particular codons (UCA and CAU) does correlate negatively with a cell's fitness, potentially due to sequestration of the corresponding rare tRNAs. The results further demonstrate how correlative conclusions made from observations of natural gene sequences can be complemented by synthetic genes, allowing decoding of the sequence features that govern the efficiency of translation and its costs.

## Results and discussion

### Translation efficiency

Looking for the effects of codon usage on translation efficiency and whether the order of the codons is important, we set out to re-analyze data from the three synthetic libraries [13,14]. The original tAI value [2] is defined for an entire gene based on all its codons as:

$$tAI_{g}={\left(\prod_{k=1}^{\ell_{g}}w_{i_{k}}\right)}^{1/\ell_{g}}$$

where *l*_*g *_is the length of the gene in codons and $w_{i_{k}}$is the relative adaptiveness value of the codon defined by the *k*th triplet in the gene.

Here we refer to the *w*_*i *_value of a single codon as the codon's tAI. This measure is an approximation of the codon's translation speed, since a codon is assigned with a high tAI if the various tRNAs that translate it are at high abundance and have high affinity towards it. Besides the tAI, there are other alternative approximations for the codon's translation speed [8,15,16] (see discussion in Additional file 1). Note that all current models have approximation as their basis, necessarily introducing inaccuracies in analyses that are based on them.

To investigate the effect of regions with less than optimal codons, for each gene we defined the 'bottleneck' as a region of a fixed number of codons, *n*, where the (harmonic) mean of the codons' tAI value is minimal (the value of *n *is related to the distance between two consecutive ribosomes on the mRNA (see Materials and methods). Assuming the codon's tAI value is an approximation for the translation speed, then 1/tAI can be regarded as the codon's translation time and the bottleneck is the region with the longest average translation time.

The bottleneck of each gene is characterized by two parameters: the location of the bottleneck - that is, number of codons from the ATG in which it occurs - and the 'strength' of the bottleneck - the average time to translate all the codons within it. To allow comparisons between the different genes and libraries below, we refer to the relative, rather than absolute, form of these variables - the relative location of the bottleneck is its location divided by the length of the gene, and the relative strength is the strength divided by the average strength (that is, the time it takes to translate the bottleneck regions divided by the total time of translation of the mRNA, or 1/tAI of the entire gene).

**Figure 1:**
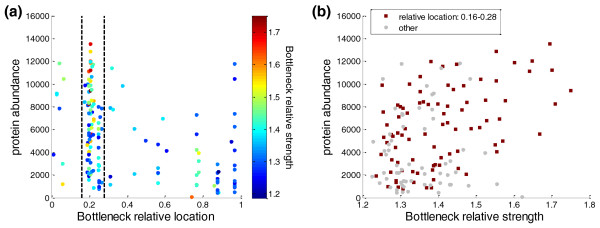
**Protein abundance versus relative location and strength of the bottleneck in the GFP library**. **(a) **All the genes in the GFP library. The x-axis is the relative location of the bottleneck in every gene; the y-axis is the per-cell protein abundance. The color of each dot is the relative strength of the bottleneck in every gene. Eighty-six of the genes are located between the two black lines that correspond to relatively early bottlenecks - that is, relative location between 0.16 and 0.28. **(b) **The correlation between the bottleneck relative strength and per-cell protein abundance for all the genes in the GFP library. The 86 genes that have a relative location between 0.16 and 0.28 are plotted as red squares, and the rest of the genes are plotted as grey circles.

We first analyzed 154 synthetic GFP genes in a library constructed by Kudla *et al. *[13]. All the synthetic GFP variants had the same amino acid sequence but different codon sequences. For these genes we calculated the bottleneck parameters using a window of length *n *= 21 codons. Note that there is uncertainty regarding the exact value of this parameter (see Materials and methods); however, experimentation with other window sizes in the range 14 <*n *< 30 did not affect results qualitatively (not shown). Figure 1a shows the relative location of the bottleneck of all GFP genes versus the protein abundance of each translated gene (see Materials and methods). The relative location is anti-correlated to the protein abundance (Pearson correlation -0.43, *P*-value 3.4 × 10^-8^; Spearman correlation -0.46, *P*-value 2.8 × 10^-9^), indicating that genes that have the bottleneck closer to the ATG (designated here as the 'proximal bottleneck') tend to have higher protein abundance levels compared to genes whose bottleneck are located towards the 3' end of the gene (designated the 'distal bottleneck').

As for the relative strength of the bottleneck, when examining the entire library of 154 genes we found a modest yet significant correlation with the protein abundance (Pearson correlation 0.38, *P*-value 1.9 × 10^-6^; Spearman correlation 0.31, *P*-value 1.2 × 10^-4^); that is, genes with long dwell times of the ribosome in the bottleneck regions tended to have higher expression levels. However, as seen in Figure 1b, this correlation is mainly contributed by genes that have a proximal bottleneck. Focusing on 86 of the genes with a proximal bottleneck (located between relative positions 0.16 to 0.28) a significant positive correlation emerged between the relative strength and the protein abundance (Pearson correlation 0.47, *P*-value 3.9 × 10^-6^; Spearman correlation 0.44, *P*-value 2.1 × 10^-5^). From Figure 1a it is seen that there are relatively few genes with a distal bottleneck that also have a similar relative strength; therefore, the influence of the relative strength on distal genes cannot be deduced.

Summarizing the analysis of the GFP library, the distribution of the codons along the transcript appears to affect the final GFP levels in the cell. A region of less efficient codons at the beginning of a transcript - for example, a proximal bottleneck - seems to enable higher protein levels. For genes with a proximal bottleneck it is also beneficial to have a relatively long dwell time of the ribosome, that is, a strong enough bottleneck. From this library we were not able to learn about the significance of the bottleneck strength in the case of genes with distal bottlenecks; however, other libraries with different distributions of bottlenecks can shed light on the question.

**Figure 2:**
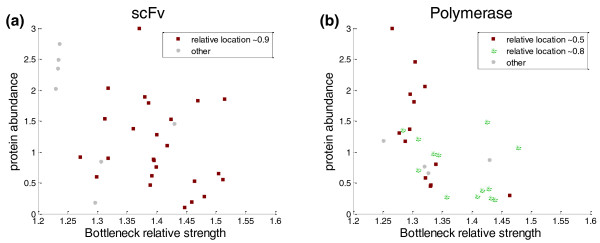
**Protein abundance versus relative strength of the bottleneck for data from the scFv and polymerase libraries**. **(a) **All the *scFv *genes; **(b) **all the polymerase genes. In both panels the x-axis is the relative strength of the bottleneck, the y-axis the per-cell protein abundance. Genes with bottlenecks at different relative locations are marked by different colors (see legend) to show the correlation between relative strength and protein abundance for genes with the same bottleneck location.

In another recent paper, by Welch *et al. *[14], two different proteins were synthesized: the DNA polymerase of Bacillus phage and an antibody fragment (scFv). For each protein there are approximately 40 different sequences in which the amino acid was kept the same while changing the codon sequence. For both proteins, the location of the bottleneck is quite far from the ATG in most synthetic variants (relative distance of approximately 0.5 and higher; Figure S1 in Additional file 2), excluding the possibility of examining the effect of the proximal bottleneck on the expression of these two proteins. Nonetheless, we could still compute the correlation between the bottleneck's parameters and protein abundance. Although less significant than in the case of the GFP library, both libraries showed an anti-correlation between protein abundance levels and the relative location of the bottleneck (Spearman correlation -0.34 (*P*-value 0.06) and -0.40 (*P*-value 0.03); Pearson correlation -0.34 (*P*-value 0.06) and -0.16 (*P*-value 0.40) for the scFv and the polymerase, respectively). Similar to the GFP library, such negative correlation indicates that proximal bottlenecks are often associated with higher expression levels. As was done for the GFP library, we looked at the correlation between protein abundance and the bottleneck relative strength (Figure 2) for specific locations, chosen based on Figure S1 in Additional file 2 (for correlations see Table S1 in Additional file 1). Interestingly, while in the case of the GFP library a proximal bottleneck became more effective with increased relative strength, in the cases of scFv and the polymerase, which featured a distal bottleneck, the strength actually showed the opposite correlation; that is, genes with long dwell times in the bottleneck regions showed lower protein abundance (Spearman correlation -0.43 (*P*-value 0.02) and -0.67 (*P*-value 7.1 × 10^-5^) for all genes of scFv and the polymerase, respectively). It is our understanding that a proximal bottleneck can have beneficial effects on protein production [5]. The bottleneck can delay the translating ribosome, causing a ribosome backlog (when in polysome), and can also reduce the density of the ribosome downstream. A proximal bottleneck minimizes the number of jammed ribosomes, thus reducing ribosome sequestering and collisions, two potential causes for a decrease in protein production. Assuming the bottleneck reduces the density of ribosomes downstream, a slower bottleneck (that is, a bottleneck with increased relative strength) will reduce even more downstream ribosome collisions, improving protein production, as seen with the GFP library. On the other hand, a distal bottleneck at the end of the ORF causes a long backlog, with no beneficial effects on expression levels. Since a bottleneck at the end of the ORF seems to have mainly negative effects on the protein translation rate, reducing its relative strength is beneficial, as seen in the case of the scFv and the polymerase.

**Figure 3:**
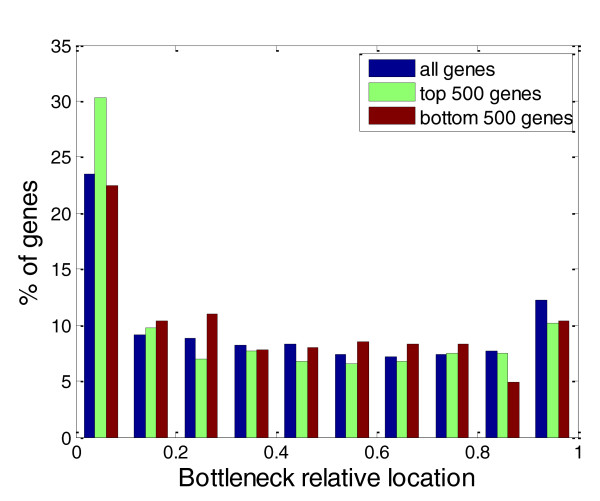
**Distribution of bottleneck relative locations for *E. coli *genes**. The distribution is shown for three groups of *E. coli *genes: all genes (blue); highly expressed genes (green); and lowly expressed genes (red). For all groups only genes longer than 100 codons are shown (this cutoff retains 90% of the *E. coli *genes). This resulted in 442 highly expressed genes (out of the top 500) and 473 lowly expressed genes (out of the bottom 500).

To further verify our assumption that the bottleneck may have beneficial effects on protein abundance when they are located at the beginning of a gene, we looked at the distribution of locations of the bottleneck in natural *Escherichia coli *genes [Refseq: NC_012947] (Figure 3; Figure S2 in Additional file 2). Indeed, for most genes with a bottleneck of high relative strength (higher than 1.3), the bottleneck region is located in the first quadrant of the transcript (relative location smaller than 0.25). For 41% of genes with a bottleneck of high relative strength, the bottleneck is located in the first quadrant (hyper-geometric significant enrichment *P*-value 6.2 × 10^-9^) and only 22% of these genes have the bottleneck located in the fourth quadrant, which is a significant depletion (hyper-geometric *P*-value 1 × 10^-4^). Examining highly expressed genes separately (see Materials and methods; Figure S2b in Additional file 2), we also observe a depletion of a strong bottleneck in the fourth quadrant (18% of the genes, hyper-geometric *P*-value 0.02) and enrichment in the first quadrant (49%, *P*-value 0.005). In contrast, a separate examination of lowly expressed genes (Figure S2c Additional file 2) reveals no significant depletion or enrichment (depletion in the fourth quadrant 18% (*P*-value 0.39); enrichment in the first quadrant 41% (*P*-value 0.15)).

Kudla *et al. *[13] showed that the folding energy of the mRNA near the initiation site influences translation rate. It was suggested that a weak secondary structure enables the ribosome to bind more quickly to the mRNA, thus enabling a faster translation rate. These observations raised the possibility that the correlation we observe between bottleneck location and protein abundance in the GFP library is due to the confounding effects of mRNA secondary structure stability. We thus carried out correlation analysis to verify that the correlations we found still hold even when examining gene sets with similar mRNA folding energy. We calculated the partial correlation between bottleneck parameters and per-cell protein abundance while controlling for the folding energy. Both the relative location correlation (Pearson correlation -0.24, *P*-value 0.004; Spearman correlation -0.27, *P*-value 9.5 × 10^-4^) and the relative strength at locations 0.16 to 0.28 (Figure 1) correlation (Pearson correlation 0.3, *P*-value 0.006; Spearman correlation, 0.24, *P*-value 0.024) remained significant even after controlling for the folding energy, indicating that bottleneck parameter correlations are significant on their own. Therefore, although in the GFP library the folding energy significantly affects the protein abundance, bottleneck location and strength also contribute to the changes in protein levels.

### The cost of production

For efficient translation we are interested not only in the levels of expressed protein from a gene but also in the cost of expression. Considering the cost of production, we looked at how introducing a new gene into the host cell influenced cell fitness. The influence on fitness is, in general, a combination of the benefit the protein provides with the burden its production puts on the system. However, assuming that the genes from the heterologous libraries discussed here do not contribute to the fitness of the host cell, the fitness decline due to expression reflects only the pure cost of production.

**Figure 4:**
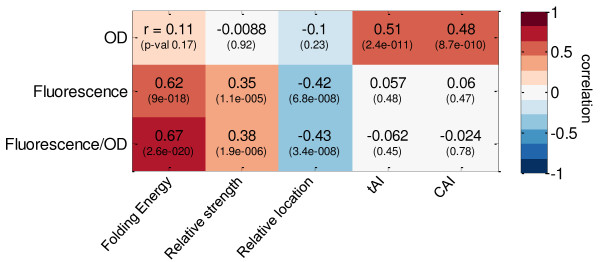
**Correlation between the GFP experimental measurement and transcript calculated parameters**. On the x-axis are different parameters that can be calculated from the transcript: folding energy of the initiation site calculated in Kudla *et al. *[13], bottleneck parameters, CAI and tAI. On the y-axis are the optical density (OD) measurement, protein abundance and per-cell protein abundance. The correlation value is indicated by both the color of the box and the number. The correlation *P*-value is given in parentheses.

Kudla *et al. *[13] showed that the measured optical density (OD), assumed to be proportional to the fitness of the host cell, is highly correlated with the CAI. Further analysis showed that the tAI is also correlated with OD (Pearson correlation 0.51, *P*-value 2.4 × 10^-11^). These two similar measures describe the entire transcript and not a particular region within it. In contrast, we found that the bottleneck parameters that significantly correlate with protein abundance are not correlated with cell fitness. Thus, the factors that correlate with fitness and those correlating with protein abundance appear distinct in this library (Figure 4). It seems that while specific regions of the transcript affect protein abundance, the fitness is affected by the codon usage of the entire transcript.

**Figure 5:**
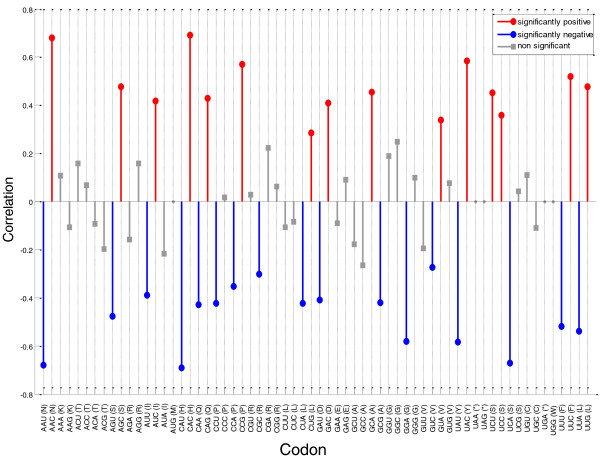
**Correlation between codon usage in a transcript and fitness**. The bar indicates the Pearson correlation value between codon frequency and OD. On the x-axis are listed all the codons in the format 'codon (amino acid)'. A correlation was determined to be significant if its *P*-value is below 0.05/61 (that is, alpha = 0.05 was corrected for the number of codons tested). Red bars represent codons for which there is a significantly positive correlation between their appearance and the OD. Blue bars represent codons that have a significant negative correlation. For codons with no significant correlation, grey squared bars are used. When no bar appears for a codon (for example AUG, UAA and so on) it means that the usage of that specific codon was constant for all genes, thus resulting in no correlation value. For usage of each amino acid in the GFP variant, see Table S3 in Additional file 1.

Trying to understand the source for the correlation between the fitness and tAI or CAI, we examined the effect of individual codons on cell fitness. We analyzed the correlation between the usage frequency of each specific codon in the GFP sequence (number of copies of the codon in the sequence) and the fitness of the cell that was expressing that GFP variant (Figure 5). Interestingly, the extent of usage of some codons is negatively correlated with fitness, is positively correlated for others, and for the rest is not correlated with fitness. The cases of negative correlation may indicate a burden on fitness due to using particular codons. In contrast, since fitness can only decrease due to GFP expression, cases of positive correlation between codon usage in a gene and its host fitness likely reflect an artificial negative correlation of synonym codons; that is, the preference for not using its alternative codons rather than a preference for expressing the codon itself.

Thus, focusing on the codons that correlate negatively with fitness, we detected three codons whose usage correlates most significantly: CAU (Pearson correlation -0.69, *P*-value < 10^-324^); AAU (Pearson correlation -0.68, *P*-value < 10^-324^); and UCA (Pearson correlation -0.67, *P*-value < 10^-324^) (Figure 5; Table S2 in Additional file 1). Further examination reveals inter-dependencies between the usage of some of these codons; in particular, the frequencies of CAU and AAU are highly correlated (r = 0.92, *P*-value 10^-64^) among themselves (the reasons for internal correlation may have to do with GFP construction methods; see Kudla *et al. *[13]). Using partial correlation analysis between the usage of each codon, we identified UCA and CAU as the main codons contributing to the decrease in the fitness (see Materials and methods).

The number of occurrences of the UCA codon, encoding serine, in a single gene varies between zero to three appearances. This codon is the rarest out of the six serine codons in the *E. coli *genome [Refseq: NC_012947], though it is not extremely rare (12.2% of all serine codons, and 0.7% of all 61 codons in the ORFs of the genome; Table S2 in Additional file 1). However, in the transcriptome (that is, the genome, weighted by the mRNA expression level from each gene; see Materials and methods) UCA is one of the rarest codons (8.7% of all serine codons and 0.45% of all 61 codons). The UCA codon is exclusively translated by the tRNA_UGA _[17]. The genome of *E. coli *has only one copy of this tRNA gene and, reassuringly, it was shown that a shortage of this tRNA decreases cell fitness [18]. The negative correlation between the copy number of the UCA codon and the fitness can thus imply that increased usage of the UCA codon causes a shortage of the corresponding tRNA, causing a decrease in fitness. Regarding codons CAU and AAU, they are negatively correlated with fitness (and with one another) yet we found no apparent reason for this.

Shortage of tRNAs explains some of the correlations between the usage of certain codons and fitness; however, it is not clear through which mechanism a shortage of tRNAs affects the fitness. The extensive usage of codons that correspond to rare tRNAs can affect the fitness in at least one of two alternative ways: by 'consuming' the tRNAs and sequestering them from participating in the translation of other transcripts; or through the unavailability of ribosomes that are delayed for longer times while searching for rare tRNAs. A simple means to distinguish between these two alternative options is to examine whether not only the number but also the location of such rare codons affects fitness. In particular, we expect that if the fitness-reducing effect of the rare codons is the jamming of ribosomes, then their utilization will be particularly harmful when located distally, closer to the 3' end of the transcript. In contrast, if the fitness-reducing effect is predominantly due to the consumption of rare tRNAs, then it is not expected to show such location dependence. In reality, we observed no correlation with the location (Figure S3 in Additional file 2), suggesting that it is the consumption of the rare tRNAs, in this case, that compromises fitness.

## Conclusions

As shown, a proximal and strong bottleneck is correlated with an increase in protein abundance. A proximal bottleneck can reduce the number of jammed ribosomes on a transcript. Therefore, it can reduce both the number of occupied ribosomes and the number of delayed ribosomes. Delaying ribosomes on the mRNA might increase their abortion rate, thus causing early termination of the translation [19], reducing protein levels. For ribosomes to jam, a fast initiation rate is required. This is usually the case in highly expressed genes, in cases of heterologous gene expression, and in synthetic libraries such as discussed here where high protein levels are desired. Due to amino acid sequence constraints for some genes, a naïve approach, using only optimal codons, might result in an unintentional distal bottleneck.

While the bottleneck parameters are correlated with protein abundance, they are not correlated with fitness. This suggests that while the occupation of more ribosomes sequesters them from the cell's pool, for most genes in the GFP library it does not cause a shortage of ribosomes, enabling the cell to continue translating other transcripts. The decrease in fitness is correlated with the increased usage of codons UCA and CAU, suggesting a shortage of the complementary tRNAs.

Our results thus show that, along with mRNA stability, codon choice does affect translation efficiency, and that naïve averaged measures such as CAI and tAI do not capture this regulatory capacity. The results also show that while codon choices do affect both translation efficiency and cell fitness, different aspects of codon selection affect differently the production capacity and costs. One direct conclusion from our results relates to the popular usage of 'His-tags', chains of histidine residues at carboxyl termini of genes in heterologous expression systems [20]. When using carboxy-terminal His-tags in bacterial expression systems it would be advantageous to encode histidine with CAC rather than with CAU for two reasons: first, because CAU appears to correlate negatively with fitness; and second, in order to avoid a bottleneck towards the end of the gene.

When trying to understand the cell system, one realizes its processes are regulated on many different levels. As shown in this paper, synthetic gene libraries enabled us to control for a significant portion of gene variability and focus on the effects of regions with less than optimal codons (the bottleneck). Identification of bottleneck effects in synthetic genes thus completes Tuller *et al*.'s [5] bioinformatics work that identified clustering of low efficient codons at the beginning of ORFs of natural genes. The results further demonstrate how correlative conclusions made from observations of natural gene sequences can be complemented by synthetic genes, allowing decoding of the sequence features governing the efficiency of translation and it costs.

It is our belief that through carefully designed synthetic libraries many other regulation processes can be understood, thus completing the first step towards understanding the regulation process as a whole.

## Materials and methods

### Defining the bottleneck

The bottleneck is a region on a gene where the harmonic mean of its codons' tAI values is minimal. For all codons except CGA, the tAI values were calculated using dos Reis *et al.*'s s-values [2]; for codon CGA the value 0.1333 was used. This codon is translated with tRNA_ACG_; however, the s-value for this interaction is very high, resulting in a very low tAI value. This tAI value is smaller by at least an order of magnitude than the smallest tAI value, causing all other codons to have a relatively high tAI, disabling this analysis. Since CGA is actually translated by tRNA_ACG_, we decided to change the s-value of this interaction to a more reasonable value, resulting in the above mentioned tAI value. Given the tRNA repertoire of *E. coli*, this change affects only the tAI value of codon CGA.

A codon tAI value is assumed to be proportional to the speed of the codon's translation [5]; higher tAI values correspond to high tRNA abundance and affinity, thus faster translation. A harmonic mean of speeds is simply an arithmetic mean of the corresponding times. Hence, looking for the region with the minimum harmonic mean of speed is equivalent to looking for the region that takes the longest time to translate.

For each region the harmonic mean of speed is:

n∑c∈Region1tAIc

where *n *is the region size, and *c *is the set of all the codons in the region (*n *codons).

To find the bottleneck, a sliding window of length *n *over the gene was used. The harmonic mean was calculated for each window and the window with the minimum value was identified. It should be noted that since we are averaging the translation time in a window, an incorrect window size might in some cases result in incorrect identification of the bottleneck. For example, if our estimated window size is too big, it might mask a cluster of a few slowly translated codons, of a more relevant size, that are surrounded by relatively rapidly translated codons. In most cases, however, the slow region is significant enough and its identification is not too sensitive to window size. Indeed, as mentioned in the Result and discussion section, our results did not change qualitatively for window sizes in the range 14 <*n *< 30.

### The bottleneck window size (*n*)

Under a maximal density scenario (fast initiation rate), the distance between two consecutive ribosomes will be minimal. In this case, when two ribosomes are translating the same mRNA simultaneously, the minimum possible distance between the two translated codons (one by each of the ribosomes) is one ribosome size (*H *codons) (Figure S4 in Additional file 2). At any given moment during the translation process, two adjacent ribosomes would have translated exactly the same codons apart from the last *H *codons - the first of the two ribosomes has already translated them, and the second is just about to start them. If the time it took the first ribosome to finish translating the *n*th codon, *T*(*n*,1), is longer than the time it takes the second ribosome to translate the *n-H*th codon, *T*(*n *- *H *,2), the second ribosome will 'bump' into the first one. That is, if *T*(*n*,1) >*T*(*n *- *H *,2), a traffic jam will be created. *T*(*n*,1)can be found by summing the time it takes the ribosome to assemble on the ATG (*B*) with the time it takes to translate the *n *codons:

$$T(n,1)=B+\sum_{i=1}^{n}t(i)$$

where *t*(*i*) is the time it takes to translate the *i*th codon. The second ribosome gains access to the ATG only when enough codons (minimum *H*) are cleared after being translated by the first ribosome. As a result a traffic jam will be created if *Tw *(*k*,*H*) >*Tw (*1*,H)+B*, where *Tw *(*k*,*H*) is the time to translate *H *consecutive codons starting from codon *k*:

$$Tw(k,H)=\sum_{i=k}^{k+H-1}t(i)$$

Therefore, the region of *H *codons with maximum translation time $\left(\underset{k=1:\text{mRNA length}-H}{\arg\max}\left(Tw(k,H)\right)\right)$ determines whether and where a traffic jam will be created (for a detailed calculation, see page 2 of Additional file 1). Choosing *n *in our bottleneck equation to be equal to *H*, it is easy to see that our bottleneck is related to this maximum.

As can be seen from this analysis, the minimal distance between two ribosomes should determine our window size. The footprint of the ribosome, which is the actual protection of the ribosome from RNA degradation, was determined quite accurately to be ten codons [21]. Due to the structure of the ribosomes, we assume that there should be some space between two consecutive 30S subunits. As a result, although only ten codons are protected, the minimal distance between the two ribosomes should be larger. Therefore, we chose to adopt the average ribosome-to-ribosome distance measured by Brandt *et al. *[22]. They measured the mean distance between the center of mass of two ribosomes on actual bacterial polysomes to be 21.6 nm [22], which is about 21 codons (0.34 nm per base). In this paper, *n *was set to be equal to *H*; that is *n *is set to 21 codons.

### The bottleneck parameters

A bottleneck is characterized by two parameters: its 'location' and its 'strength'.

The 'location' of the bottleneck is defined as the location in the gene of the bottleneck's first codon (*k *codons from the ATG). The relative location of the bottleneck is defined as the location of the bottleneck divided by the number of possible windows; for example, $\frac{k}{l-n+1}$, where *k *is the location of the bottleneck, *l *is the length of the gene, and *n *is the window size.

The 'strength' of the bottleneck is defined as the arithmetic average of 1/tAI values for the codons in the region, for example, 1n∑c∈Region1tAIc (the inverse of the harmonic mean). The relative strength of the bottleneck is defined as the strength of the bottleneck divided by the average 1/tAI for the entire gene, for example, $\frac{\frac{1}{n}\sum_{c\in bottleneck}\frac{1}{tAI_{c}}}{\frac{1}{l}\sum_{c=1}^{l}\frac{1}{tAI_{c}}}$; where *l *is the number of codons in the gene (excluding the stop codon).

### Per-cell protein abundance

To get an estimate for protein expression per cell from the GFP library data [13], we normalized the measured protein abundance (measured by OD), which serves here as a proxy for the population size, the OD. The protein abundance levels for the data from Welch *et al. *[14] were measured while keeping the OD constant. Therefore, we can use this protein abundance as an already normalized protein level per cell.

### Highly and lowly expressed genes of *E. coli*

The *E. coli *mRNA levels were taken from Lu *et al. *[23]. The highly expressed genes are the top 500 genes, and the lowly expressed genes are the bottom 500 genes (genes with no mRNA recorded were ignored). However, for both groups only genes that are longer than 100 codons were used.

### Finding the main anti-correlated codons

We used partial correlation to find the codons that contribute the most to the decrease in cell fitness. The highest contributors were filtered according to the following steps. First, find codons that have a negative correlation to the OD (29 codons). We were looking for codons that caused a decrease in the fitness; hence, only anti-correlated codons. Second, for all codons left, we calculated the partial correlation matrix M(*i,j*) = Partial correlation (codon *i*, OD | codon *j*). Third, find the minimum absolute value of the partial correlation for each codon and rank the codons in a descending order accordingly. This gives us the codons with a correlation that cannot be explained by correlation to other codons (see Table S4 in Additional file 1 for a list of all codons with *P*-value < 0.1).

The codon at the top of the list is UCA, which is anti-correlated to the OD and its correlation cannot be explained by other codons. The second contributing codon is CAU, which has the highest partial correlation (-0.36, *P*-value 8.5 × 10^-6^) when controlling for the UCA codon. This codon is also the second codon in the ranked list. All other codons have a partial correlation < 0.2 with a *P*-value ≥ 0.04 when controlling with one of the two codons (either UCA or CAU).

### Calculating codon usage in the genome

The genome for *E. coli *strain B21 (which was used by Kudla *et al. *[13]) was downloaded from the NCBI ([Refseq:NC_012947], 11 January 2010)]. For each codon we counted its appearance in all the ORFs and normalized by the total number of codons.

### Calculating codon usage in the transcriptome

mRNA levels were taken from Lu *et al. *[23]. If a gene did not have a measurement, it was assumed to have a zero mRNA level. The measurements were done with *E. coli *strain K12 MG1655; thus, the sequence used for the calculation was different from that used for genome codon usage. The sequence was downloaded from NCBI ([Refseq: NC_000913], 1 April 2010). The contribution of each gene was calculated by multiplying the mRNA level measurements for the gene by the codon usage of the same gene. The contributions of all genes were summed for each codon and then divided by the total sum of all codons.

## Abbreviations

CAI: codon adaptation index; GFP: green fluorescence protein; OD: optical density; ORF: open reading frame; tAI: tRNA adaptation index.

## Authors' contributions

SN carried out all analyses. SN and YP conceived the work, analyzed the data and wrote the paper.

## Supplementary Material

Additional file 1 **Supplementary methods**. This file includes a discussion regarding codon translation speed, additional tables not included in the main text, and figure legends for the supplementary figures in Additional file 2. Click here for file

Additional file 2 **Supplementary figures**. Additional figures not included in the main text. Click here for file
