# Supplementary material for: The role of codon selection in regulation of translation efficiency deduced from synthetic libraries
Source: Genome Biol. 2011 Feb 1;12(2):R12. doi: 10.1186/gb-2011-12-2-r12 (PMC3188794; doi:10.1186/gb-2011-12-2-r12)
Supplement: Additional file 1 — Supplementary methods. This file includes a discussion regarding codon translation speed, additional tables not included in the main text, and figure legends for the supplementary figures in Additional file 2. [file gb-2011-12-2-r12-S1.DOC]

# Supplement Text

## Codons translation speed

In this paper we used the tRNA adaptation index (tAI) developed by dos Reis [2] as an approximation for the codon translation speed. The tAI index was developed mainly based on optimizing the translation efficiency of highly express genes. Two recent papers [15, 16] used evolutionary selection for translation efficiency as a means to learn the efficiency and coefficients of codon-anticodon interactions. They found that some non-standard combinations seem to be selected for and hence deduced to be translated at high rates. One of the major difference between their conclusion to the tAI is the value of the U:U interaction (codon with wobble-U translated by a tRNA with a wobble- U) for amino acid in a 4-box. In the tAI calculations this interaction was assumed to be forbidden (sU:U=1) while based on selection it was suggested that this interaction is quite potent, which should result in a low sU:U in the tAI model.

To investigate the U:U interaction effects we manually added the sU:U interaction to the tAI calculations at 4-box cases and scanned the effects over the full possible range of values of sU:U, form 0 to 1. First we looked how adding sU:U changed the bottleneck parameters. Figure S5 shows the correlation between our original bottleneck parameters and the newly calculated one. As expected the correlation decreases as sU:U decreases. The correlation decreases from 1 for sU:U=1 (which is as in the original dos Reis tAI values) to 0.61 for sU:U=0, for the bottleneck relative location and 0.51 for the bottleneck relative strength.

Next we looked how the modified tAI predicts expression levels of the *E. coli* mRNA levels [26] for 3 different groups: all genes, highly expressed (top 250) and lowly express (bottom 250). As can be seen in Figure S6 for all genes and the highly expressed ones the correlations increase with an introduction of the U:U interaction (sU:U<1). However it seems that the main contribution to the increase occurs merely by introducing the interaction, i.e. reducing sU:U from 1 to 0.7 results in half of the increase in the correlation. For this graph we find it hard to deduce what should be the optimal sU:U value, but the result indeed suggests a desired correction for the tAI.

Finally we look at the two main correlations we find in the GFP library: the correlation with between the bottleneck relative location to the protein abundance and the correlation between the bottleneck relative strength for genes with the same relative location (between 0.16-0.28) and protein abundance. For both correlations we see (figure S7) that the introduction of sU:U actually reduces the correlations.

## The bottleneck window size (*n*) - detailed calculation

The bottleneck is the region that will have the most slowing down effect on the ribosome. This region will only have a bottleneck effect if it slows the ribosomes enough to affect consecutive ribosome. As explained in the following the size of this region has to be about the minimum ribosome-to-ribosome distance (denoted below as *H*).

We make first the following definitions:

1. Traffic jam will be caused if the time it takes the first ribosome to finish translation of the nth codon is longer than the time it takes the second ribosome to finish translation of codon n-H. In this case the second ribosome will not be able to proceed to the (n-H+1)-th codon since it will collide with the first ribosome and hence will be delayed.
2. A ribosome can start assembling on the ATG when the first H codons (the minimum distance required between 2 ribosomes) are cleared from the preceding ribosome. Let B be the time it takes the ribosome to bind and assemble on the ATG.
3. If *t(i)* is the time it takes to translate codon *i,* and *T(n,j*) is the time it takes the j^th^ ribosome to finish translation of the *n*^th^ codon, where *n* is the codon at the leading edge of the ribosome.

then:

- 1. The time it takes the first ribosome to reach the *n*^th^ codon:
  2. The time it takes the second ribosome to reach the (n-H)-th codon:

1. For a traffic jam to be created, the time for the first ribosome to finish translation of the *n*^th^ codon should be longer than the time it takes the second ribosome to finish translation of the *n-H* codon :

1. We define $\text{Tw}(k,H)$ is the time to translate *H* consecutive codons starting from codon *k* . Therefore, a traffic jam will be created if:
2. For any traffic jam to be created along the gene:

Inspecting the slowest region (of *H* codons) for the specific gene (bottleneck strength), if the time to translate that region minus the time it takes to translate the first *H* codons is longer than the time to finish “assembling” the ribosome (*B*) than traffic jam will be created.

As can be seen from the equation the region size which determine whether two consecutive ribosomes will collide has the size of the minimum distance between 2 ribosomes.

# Supplement Figures

## Figure S1: protein abundance vs. bottleneck relative location of data from Welch *et al*.’s libraries.

In the left figure (A) plotted all the *scFv* genes and in the right figure (B) plotted all the Polymerase genes. In both figures the x-axis is the gene’s relative location, the Y-axis is the per-cell protein abundance and the color is the gene’s relative strength.

## Figure S2: The distribution (2D histogram) of the bottleneck of the E. coli genes.

X-axis is the relative location, Y-axis the relative strength and the color is the % of genes having a bottleneck matching the parameters. **A** all *E. coli* genes longer than 100 (well above region size and still maintain 90% of the *E. coli* genes) codons were plotted. **B** only the highest expressed genes were plotted. The 500 genes which have the highest transcript levels were chosen and from these the genes with 100 codons or longer were taken, making a total of 442 genes. **C** only the lowest expressed genes were plotted. The 500 genes which have the lowest transcript levels were chosen and from these the genes with 100 or longer were taken, making a total of 473 genes.

## Figure S3: location of first copy of a codon vs. the fitness.

Each point represents a gene in the GFP library. **A**, plotted is the location of the first CAU codon for each GFP variant vs. the variants’ OD. In figure B, the location of the first UCA codon in the GFP variants is plotted vs. the variants’ OD.

## Figure S4: distances between two consecutive ribosomes

The figure illustrates two consecutive ribosomes, on the same mRNA, with the second one (left) currently being assembled on the ATG. The size of a ribosome in the figure is *H* codons. *H_L_* is the distance from the ribosome A-site to the left end of the ribosome, *H_R_* is the distance from the A-site to the right end of the ribosome. The illustration shows that the minimum distance between two ribosomes' A-sites is *H* which is also “one ribosome size”. It can also be seen that in order for the second ribosome to start assembling on the ATG the first ribosome should have cleared the assembly area, e.g. translate *H* codons.

## Figure S5: Correlation between the bottleneck parameters for different sU:U values

The correlation between the bottleneck relative location (**A),** and bottleneck relative strength **(B),** calculated once with the original dos-Reis tAI values and with alternative values of sU:U.

## Figure S6: The gene expression prediction quality of the modified tAI values

For each sU:U value we calculated the correlation between the gene’s tAI value to the expression level. In the figure the correlation is plotted for three different groups: all the *E. coli* genes (blue), only the highly express ones, top 250 (green) and the lowly express ones, bottom 250 (red).

## Figure S7: correlation between bottleneck parameters and the protein abundance for different sU:U values.

**A** Correlation between the bottleneck relative location and the protein abundance for all the GFP variants, for different sU:U values. The blue line is the correlation between the relative location and the abundance while the green line is the p-value of each correlation. **B** Correlation between the bottleneck relative strength and the protein abundance for the 86 GFP variants for which the bottleneck is located in between 0.16 to 0.28 (as done in the main text) for different sU:U values. The blue line is the correlation between the relative location and the abundance while the green line is the p-value of each correlation.

# Supplement Tables

## Table S1. The correlations between the bottleneck relative strength to the protein abundance for the *scFv* and Polymerase libraries.

The relative location regions were chosen to incorporate many genes with the same relative location; the regions were chosen based on the plots in figure S1.

| Protein | Relative location | Number of genes | Correlation with the  bottleneck relative strength  Pearson; Spearman | p-value  Pearson; Spearman |
| --- | --- | --- | --- | --- |
| scFv | 0.9-1 | 25 | -0.23; -0.32 | 0.27; 0.11 |
| scFv | 0-1 (All) | 42 | -0.43; -0.41 | 0.01; 0.02 |
| Polymerase | 0.48-0.52 | 13 | -0.60; -0.67 | 0.03; 0.015 |
| Polymerase | 0.76-0.82 | 13 | -0.38; -0.43 | 0.2; 0.14 |
| Polymerase | 0-1 (All) | 39 | -0.55;-0.67 | 0.0018; 7.1e-5 |

## Table S2 – Codon parameters

For each codon the tables contains it amino acid, number of copies of complementary tRNA in the genome, its tAI value, the Pearson correlation with the OD measurements, the codon usage in the genome and the in the transcriptome. Except for the transcriptome all values are based on E. coli strain B, the transcriptome was calculated for *E. coli* K12 (see methods). When NaN (Not a Number) is listed it means that a correlation cannot be calculated due to a constant value of codons for all GFP variants

| Amino  acid | Codon | # tRNA  Copies | tAI | correlation | p-value | Codon usage in genome % | Codon usage in transcriptome % |
| --- | --- | --- | --- | --- | --- | --- | --- |
| N | AAU | 0 | 0.39 | -0.68 | <E-324 | 1.73 | 1.21 |
| N | AAC | 4 | 0.67 | 0.68 | <E-324 | 2.16 | 2.69 |
| K | AAA | 6 | 1.00 | 0.11 | 1.96E-01 | 3.37 | 4.25 |
| K | AAG | 0 | 0.32 | -0.11 | 1.96E-01 | 1.02 | 1.29 |
| T | ACU | 0 | 0.20 | 0.16 | 5.71E-02 | 0.89 | 1.30 |
| T | ACC | 2 | 0.33 | 0.07 | 4.09E-01 | 2.33 | 2.60 |
| T | ACA | 1 | 0.17 | -0.09 | 2.57E-01 | 0.68 | 0.48 |
| T | ACG | 1 | 0.22 | -0.20 | 1.64E-02 | 1.45 | 1.02 |
| S | AGU | 0 | 0.10 | -0.48 | 8.13E-10 | 0.86 | 0.54 |
| S | AGC | 1 | 0.17 | 0.48 | 8.13E-10 | 1.61 | 1.34 |
| R | AGA | 1 | 0.17 | -0.16 | 5.48E-02 | 0.18 | 0.10 |
| R | AGG | 1 | 0.22 | 0.16 | 5.48E-02 | 0.11 | 0.05 |
| I | AUU | 0 | 0.30 | -0.39 | 9.18E-07 | 3.05 | 2.57 |
| I | AUC | 3 | 0.50 | 0.42 | 1.21E-07 | 2.52 | 3.26 |
| I | AUA | 0 | 0.055 | -0.22 | 8.02E-03 | 0.41 | 0.19 |
| M | AUG | 7 (3.5, 3.5)* | 0.58 | NaN | NaN | 2.81 | 2.73 |
| H | CAU | 0 | 0.10 | -0.69 | <E-324 | 1.28 | 0.95 |
| H | CAC | 1 | 0.17 | 0.69 | <E-324 | 0.98 | 1.13 |
| Q | CAA | 2 | 0.33 | -0.43 | 5.75E-08 | 1.53 | 1.15 |
| Q | CAG | 2 | 0.44 | 0.43 | 5.75E-08 | 2.89 | 2.93 |
| P | CCU | 0 | 0.10 | -0.42 | 8.35E-08 | 0.69 | 0.57 |
| P | CCC | 1 | 0.17 | 0.02 | 8.45E-01 | 0.55 | 0.29 |
| P | CCA | 1 | 0.17 | -0.35 | 1.08E-05 | 0.83 | 0.73 |
| P | CCG | 1 | 0.22 | 0.57 | 4.65E-14 | 2.35 | 2.54 |
| R | CGU | 4 | 0.67 | 0.03 | 7.23E-01 | 2.11 | 2.88 |
| R | CGC | 0 | 0.48 | -0.30 | 1.92E-04 | 2.22 | 2.09 |
| R | CGA | 0 | 0.13 | 0.22 | 6.21E-03 | 0.35 | 0.17 |
| R | CGG | 1 | 0.17 | 0.06 | 4.53E-01 | 0.53 | 0.25 |
| L | CUU | 0 | 0.10 | -0.11 | 1.95E-01 | 1.10 | 0.79 |
| L | CUC | 1 | 0.17 | -0.08 | 3.08E-01 | 1.11 | 0.82 |
| L | CUA | 1 | 0.17 | -0.42 | 7.56E-08 | 0.39 | 0.20 |
| L | CUG | 4 | 0.72 | 0.29 | 4.45E-04 | 5.33 | 5.80 |
| D | GAU | 0 | 0.30 | -0.41 | 2.48E-07 | 3.20 | 2.97 |
| D | GAC | 3 | 0.50 | 0.41 | 2.48E-07 | 1.91 | 2.54 |
| E | GAA | 4 | 0.67 | -0.09 | 2.79E-01 | 3.97 | 4.80 |
| E | GAG | 0 | 0.21 | 0.09 | 2.79E-01 | 1.77 | 1.80 |
| A | GCU | 0 | 0.20 | -0.18 | 3.00E-02 | 1.54 | 2.31 |
| A | GCC | 2 | 0.33 | -0.27 | 1.13E-03 | 2.57 | 2.09 |
| A | GCA | 3 | 0.50 | 0.46 | 6.25E-09 | 2.02 | 2.23 |
| A | GCG | 0 | 0.16 | -0.42 | 9.49E-08 | 3.41 | 3.17 |
| G | GGU | 0 | 0.39 | 0.19 | 2.16E-02 | 2.48 | 3.39 |
| G | GGC | 4 | 0.67 | 0.25 | 2.44E-03 | 2.99 | 3.30 |
| G | GGA | 1 | 0.17 | -0.58 | 8.66E-15 | 0.78 | 0.47 |
| G | GGG | 1 | 0.22 | 0.10 | 2.39E-01 | 1.10 | 0.71 |
| V | GUU | 0 | 0.20 | -0.20 | 1.72E-02 | 1.83 | 2.66 |
| V | GUC | 2 | 0.33 | -0.27 | 7.94E-04 | 1.52 | 1.29 |
| V | GUA | 5 | 0.83 | 0.34 | 2.50E-05 | 1.10 | 1.39 |
| V | GUG | 0 | 0.27 | 0.08 | 3.52E-01 | 2.64 | 2.36 |
| Y | UAU | 0 | 0.30 | -0.58 | 7.11E-15 | 1.60 | 1.24 |
| Y | UAC | 3 | 0.50 | 0.58 | 7.11E-15 | 1.21 | 1.46 |
| * | UAA | 0 | 0.00 | NaN | NaN | 0.21 | 0.27 |
| * | UAG | 0 | 0.00 | NaN | NaN | 0.02 | 0.01 |
| S | UCU | 0 | 0.20 | 0.45 | 8.07E-09 | 0.84 | 1.19 |
| S | UCC | 2 | 0.33 | 0.36 | 7.79E-06 | 0.86 | 1.05 |
| S | UCA | 1 | 0.17 | -0.67 | <E-324 | 0.70 | 0.45 |
| S | UCG | 1 | 0.22 | 0.04 | 6.08E-01 | 0.90 | 0.60 |
| C | UGU | 0 | 0.10 | 0.11 | 1.84E-01 | 0.51 | 0.38 |
| C | UGC | 1 | 0.17 | -0.11 | 1.84E-01 | 0.65 | 0.52 |
| * | UGA | 0 | 0.00 | NaN | NaN | 0.09 | 0.06 |
| W | UGG | 1 | 0.17 | NaN | NaN | 1.53 | 1.11 |
| F | UUU | 0 | 0.20 | -0.52 | 1.22E-11 | 2.22 | 1.54 |
| F | UUC | 2 | 0.33 | 0.52 | 1.22E-11 | 1.65 | 2.05 |
| L | UUA | 1 | 0.17 | -0.54 | 1.70E-12 | 1.38 | 0.79 |
| L | UUG | 1 | 0.22 | 0.48 | 9.38E-10 | 1.36 | 0.91 |

* Met is partly initiation tRNA and partly tRNA decoding regular Met codons. We assumed that about half of the Met tRNAs are used for initiation.

## Table S3 Amino-acid usage in the GFP sequence

For each amino acid the table lists the number of times it is used in the GFP protein.

| Amino acid | Copy number in GFP | Amino acid | Copy number in GFP | Amino acid | Copy number in GFP | Amino acid | Copy number in GFP | Amino acid | Copy number in GFP |
| --- | --- | --- | --- | --- | --- | --- | --- | --- | --- |
| A | 8 | C | 2 | H | 9 | M | 6 | T | 16 |
| R | 6 | Q | 8 | I | 12 | F | 12 | W | 1 |
| N | 13 | E | 16 | L | 21 | P | 10 | Y | 11 |
| D | 18 | G | 22 | K | 20 | S | 10 | V | 18 |

## Table S4. List of all codons which even after partial correlation still had a significant (p-value<0.1) correlation.

The codons with p-value <0.1 are:

| Codon | Minimum negative partial correlation (p-value) | Controlling codon in the partial correlation |
| --- | --- | --- |
| UCA | -0.29 (0.0004) | CAU |
| CAU | -0.24 (0.003) | AAU |
| AGU | -0.17 (0.04) | UCA |
| AAU | -0.156 (0.06) | CAU |
| GGA | -0.156 (0.06) | CAU |
| GUC | -0.15 (0.07) | UCA |
