# Supplementary figures and images for: The role of codon selection in regulation of translation efficiency deduced from synthetic libraries
Source: Genome Biol. 2011 Feb 1;12(2):R12. doi: 10.1186/gb-2011-12-2-r12 (PMC3188794; doi:10.1186/gb-2011-12-2-r12)

Figure S1

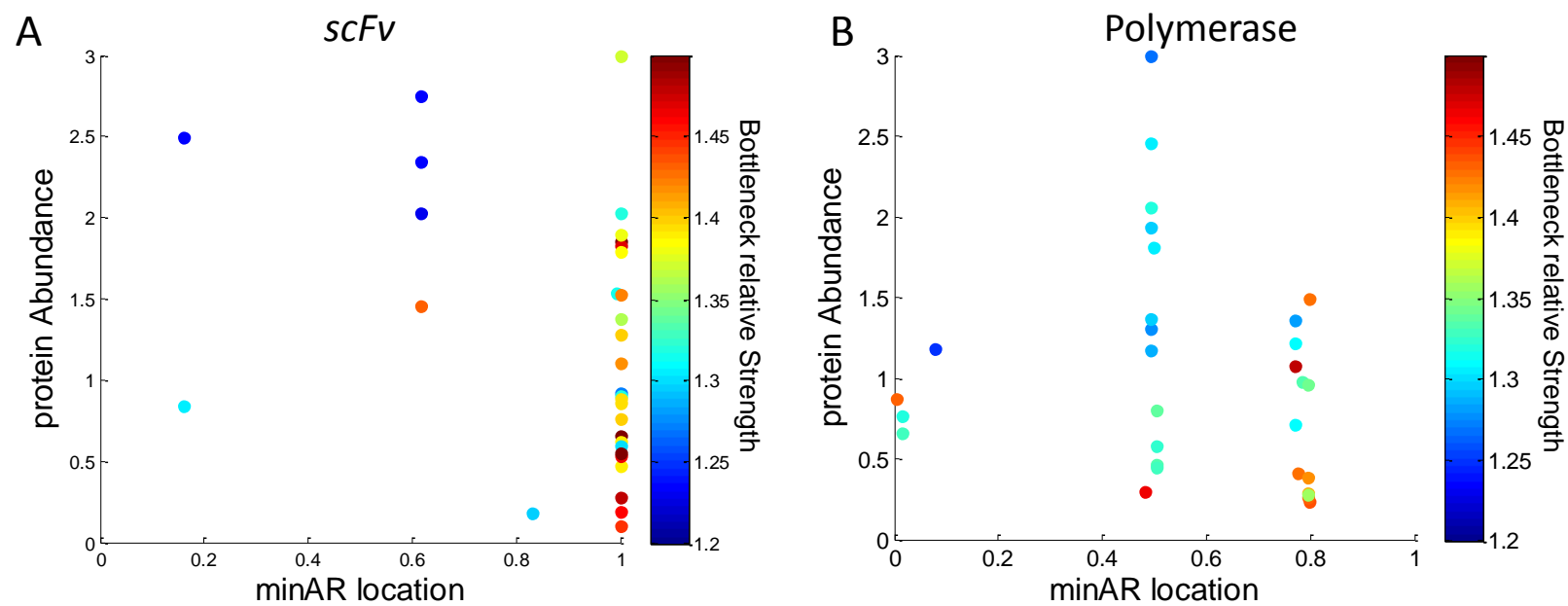

Figure S2

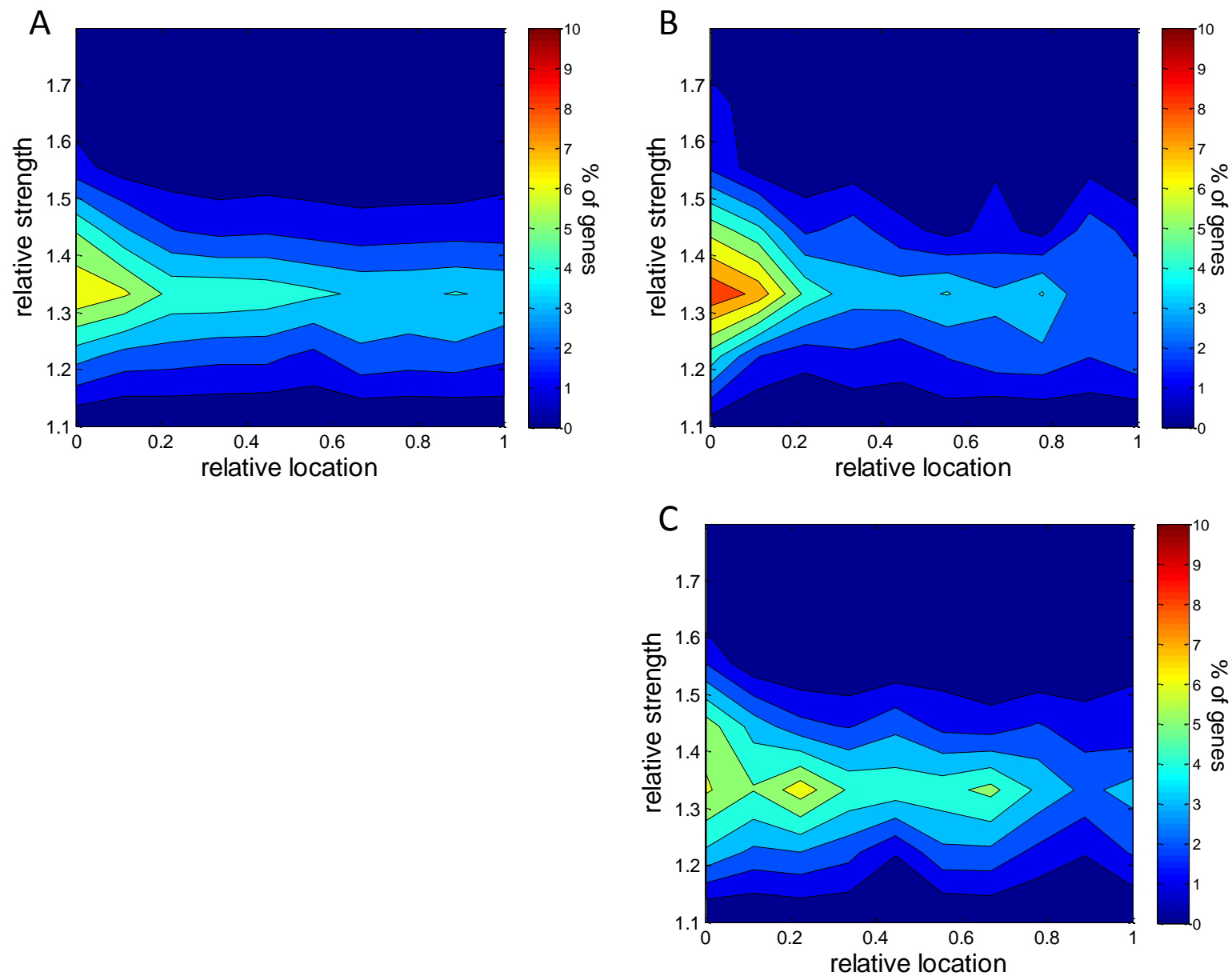

Figure S3

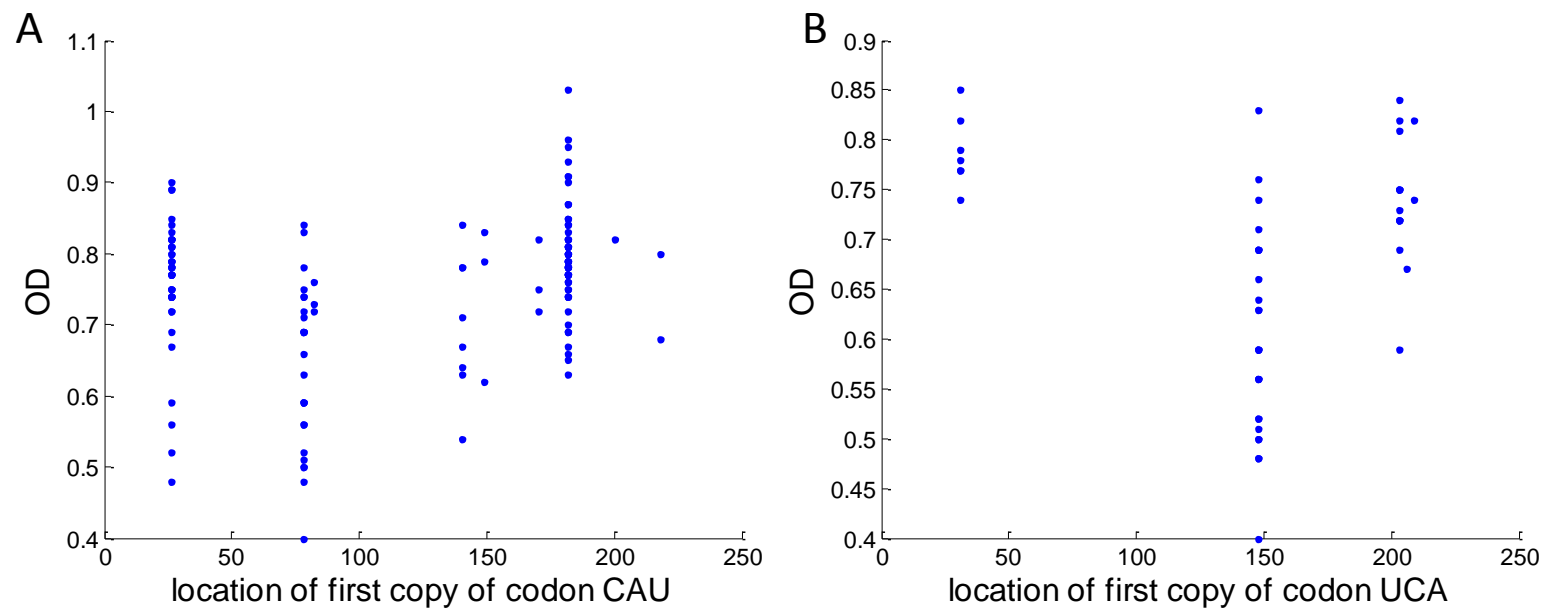

Figure S4

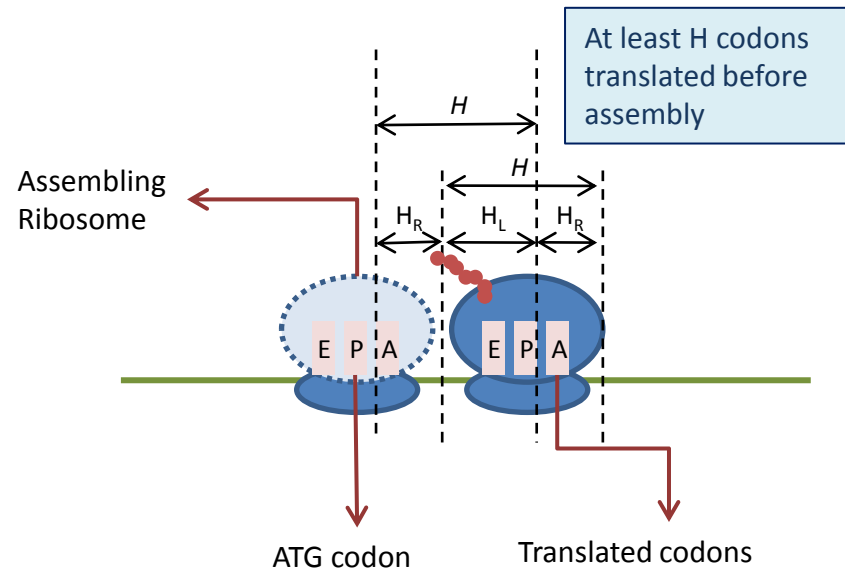

Figure S5

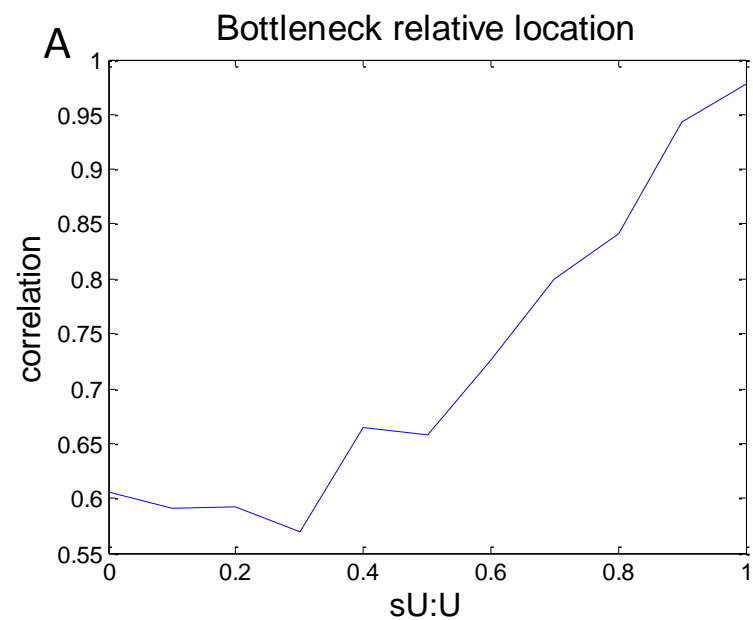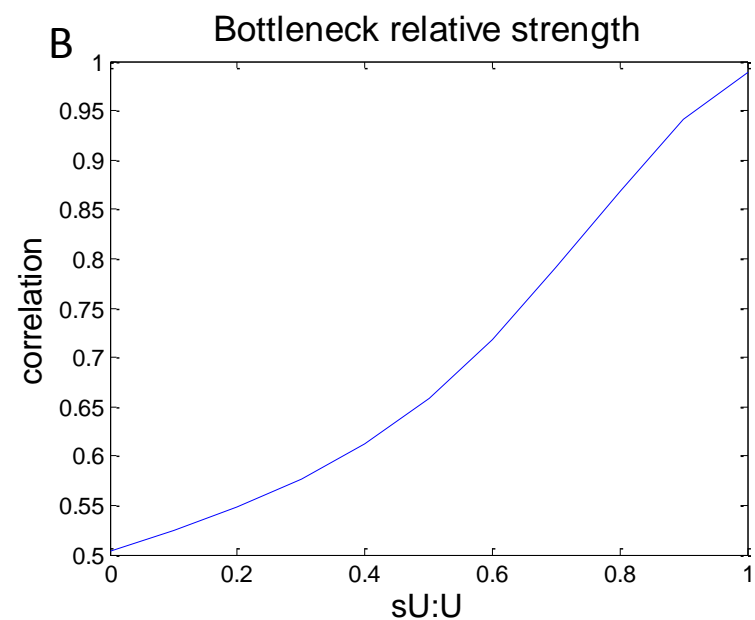

Figure S6

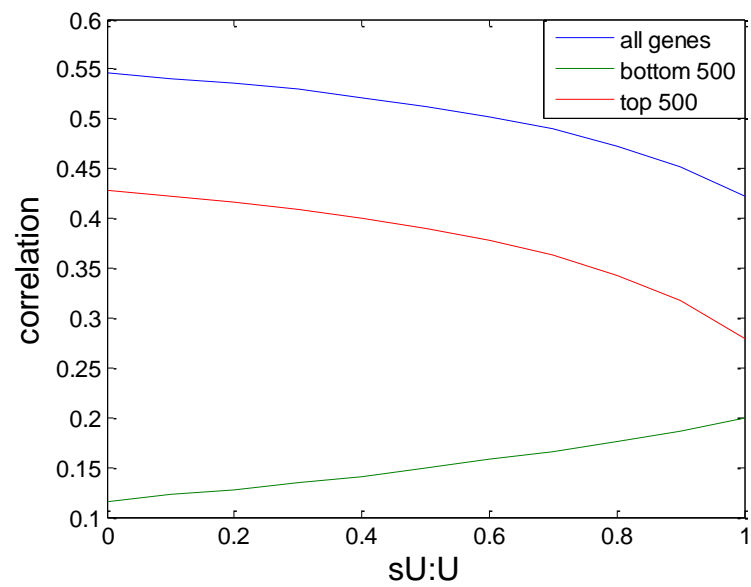

Figure S7

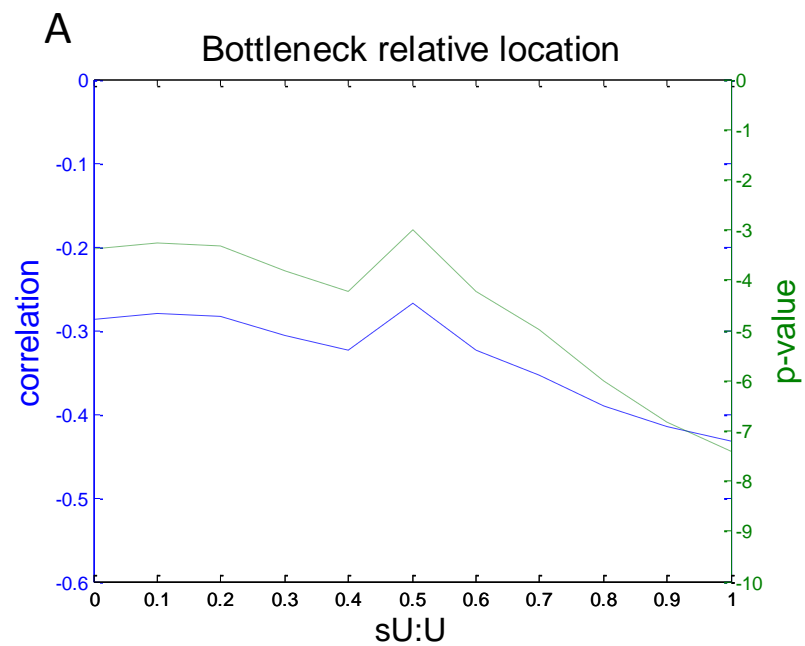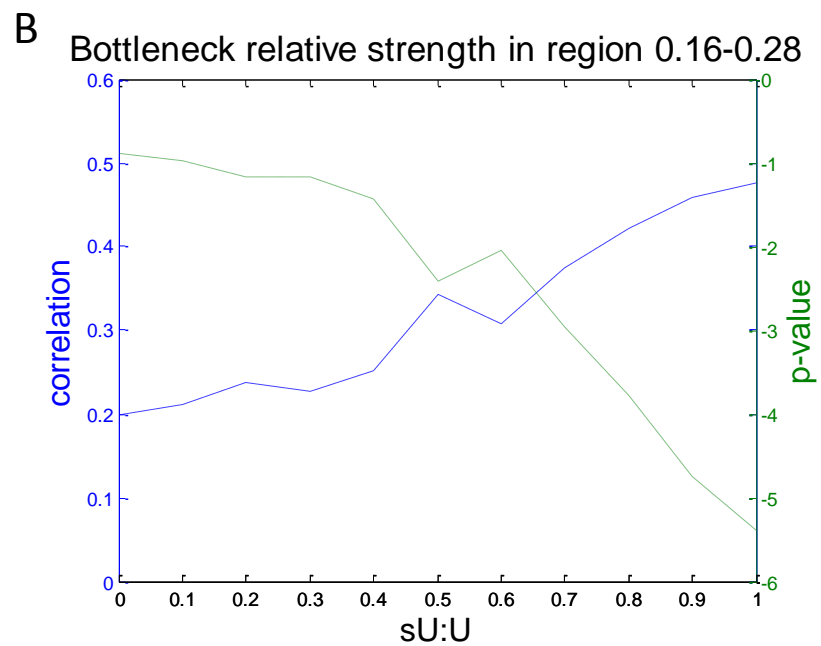

Supplement: Additional file 2 — Supplementary figures. Additional figures not included in the main text. [file gb-2011-12-2-r12-S2.PDF]
